# Supplementary material for: Comprehensive Analysis of Ferroptosis Regulators with Regard to PD-L1 and Immune Infiltration in Low-Grade Glioma
Source: Int J Mol Sci. 2023 Aug 17;24(16):12880. doi: 10.3390/ijms241612880 (PMC10454415; doi:10.3390/ijms241612880)
Supplement: Supplementary file 1 [file ijms-24-12880-s001.zip › Supplementary Figure S1.pdf]

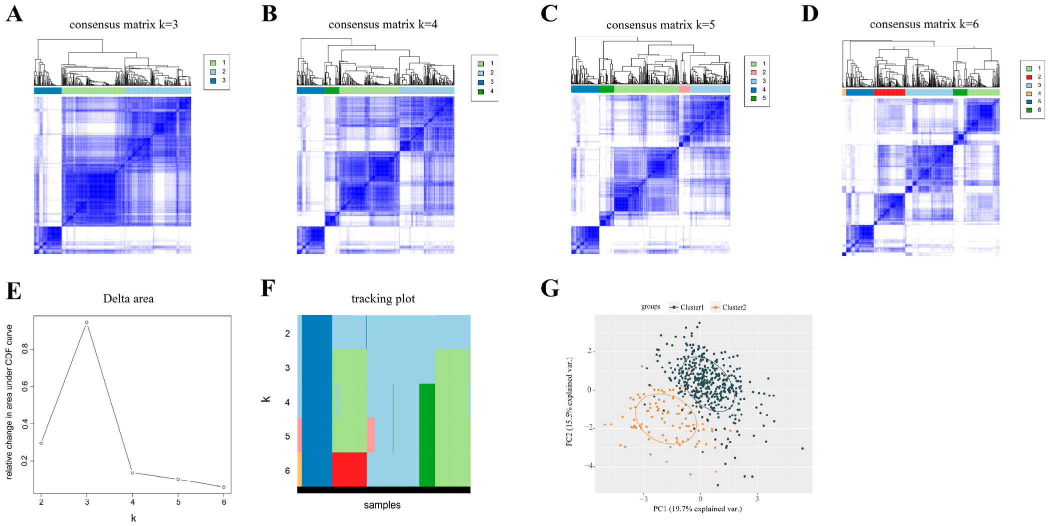

Supplementary Figure S1. Consensus clustering for FRGs in low grade glioma (LGG). (**A-D**) Heat maps exhibit the clustering matrix for FRGs in LGG patients for k = 3, 4, 5, and 6. The tighter and clearer the clusters are, the more optimal the cluster. (**E**) Delta area curve of consensus clustering for k = 2 to 6. (**F**) Tracking plot of consensus clustering for k = 2 to 6. (**G**) Principal component analysis of LGG patients' FRGs expression profiles demonstrates 2 patient clusters.
