# Supplementary material for: Comprehensive Analysis of Ferroptosis Regulators with Regard to PD-L1 and Immune Infiltration in Low-Grade Glioma
Source: Int J Mol Sci. 2023 Aug 17;24(16):12880. doi: 10.3390/ijms241612880 (PMC10454415; doi:10.3390/ijms241612880)
Supplement: Supplementary file 1 [file ijms-24-12880-s001.zip › Supplementary Table S1.pdf]

Supplementary Table S1. The oligonucleotides used in this study.

Name		Sequence (5'->3')
Beta-actin	Forward primer	CATGTACGTTGCTATCCAGGC
	Reverse primer	CTCCTTAATGTCACGCACGAT
SLC1A5	Forward primer	CTCCAGCCCTCGGGAGTAAAT
	Reverse primer	AACACTACCAAGCCCAGGATG
HSPA5	Forward primer	CACTCCTGAAGGGGAACGTC
	Reverse primer	ACCACCTTGAACGGCAAGAA
LPCAT3	Forward primer	CTGAGCCTTAACAAGTTGGCG
	Reverse primer	AGCAAAAGGTAGTGAGGACGG
NFE2L2	Forward primer	ACTACTCCCAGGTTTCTTCGG
	Reverse primer	AGTTTGGCTTCTGGACTTGGA
sh-SLC1A5-#1	Forward oligo	CCGGGCTCATACTCTACCACCTATGCTCGAG CATAGGTGGTAGAGTATGAGCTTTTTG
	Reverse oligo	AATTCAAAAAGCTCATACTCTACCACCTATG CTCGAGCATAGGTGGTAGAGTATGAGC
sh-SLC1A5-#2	Forward oligo	CCGGGCTTATCCGCTTCTTCAACTCCTCGAG GAGTTGAAGAAGCGGATAAGCTTTTTG
	Reverse oligo	AATTCAAAAAGCTTATCCGCTTCTTCAACTC CTCGAGGAGTTGAAGAAGCGGATAAGC
sh-HSPA5-#1	Forward oligo	CCGGGGTTACCCATGCAGTTGTTACCTCGAG GTAACAACTGCATGGGTAACCTTTTTG
	Reverse oligo	AATTCAAAAAGGTTACCCATGCAGTTGTTAC CTCGAGGTAACAACTGCATGGGTAACC
sh-HSPA5-#2	Forward oligo	CCGGGGGCAAAGATGTCAGGAAAGACTCGAG TCTTTCCTGACATCTTTGCCCTTTTTG
	Reverse oligo	AATTCAAAAAGGGCAAAGATGTCAGGAAAGA CTCGAGTCTTTCCTGACATCTTTGCCC
sh-LPCAT3-1	Forward oligo	CCGGGGAGGGAAAGATCAGAATTCCCTCGAG GGAATTCTGATCTTTCCCTCCTTTTTG
	Reverse oligo	AATTCAAAAAGGAGGGAAAGATCAGAATTCC CTCGAGGGAATTCTGATCTTTCCCTCC
sh-LPCAT3-#2	Forward oligo	CCGGGCAACAGAAATATGCCATACGCTCGAG CGTATGGCATATTTCTGTTGCTTTTTG
	Reverse oligo	AATTCAAAAAGCAACAGAAATATGCCATACG CTCGAGCGTATGGCATATTTCTGTTGC
sh-NFE2L2-#1	Forward oligo	CCGGGCCCATTGATGTTTCTGATCTCTCGAG AGATCAGAAACATCAATGGGCTTTTTG
	Reverse oligo	AATTCAAAAAGCCCATTGATGTTTCTGATCT CTCGAGAGATCAGAAACATCAATGGGC
sh-NFE2L2-#2	Forward oligo	CCGGGGGATATGGTACAACCCTTGTCTCGAG ACAAGGGTTGTACCATATCCCTTTTTG
	Reverse oligo	AATTCAAAAAGGGATATGGTACAACCCTTGT CTCGAGACAAGGGTTGTACCATATCCC
